# Potential of antibody–drug conjugates (ADCs) for cancer therapy

**DOI:** 10.1186/s12935-022-02679-8

**Published:** 2022-08-13

**Authors:** Hany E. Marei, Carlo Cenciarelli, Anwarul Hasan

**Affiliations:** 1 Department of Cytology and Histology, Faculty of Veterinary Medicine, Mansoura University, Mansoura, Egypt; 2 Institute of Translational Pharmacology (IFT)-CNR, Rome, Italy; 3 Department of Mechanical and Industrial Engineering, College of Engineering, Qatar University, Doha, Qatar

**Keywords:** Antibody drug conjugate, ADCs, Targeted cancer therapy, Cytotoxic drugs, Solid cancer, Hematological malignancies

## Abstract

The primary purpose of ADCs is to increase the efficacy of anticancer medications by minimizing systemic drug distribution and targeting specific cells. Antibody conjugates (ADCs) have changed the way cancer is treated. However, because only a tiny fraction of patients experienced long-term advantages, current cancer preclinical and clinical research has been focused on combination trials. The complex interaction of ADCs with the tumor and its microenvironment appear to be reliant on the efficacy of a certain ADC, all of which have significant therapeutic consequences. Several clinical trials in various tumor types are now underway to examine the potential ADC therapy, based on encouraging preclinical results. This review tackles the potential use of ADCs in cancer therapy, emphasizing the essential processes underlying their positive therapeutic impacts on solid and hematological malignancies. Additionally, opportunities are explored to understand the mechanisms of ADCs action, the mechanism of resistance against ADCs, and how to overcome potential resistance following ADCs administration. Recent clinical findings have aroused interest, leading to a large increase in the number of ADCs in clinical trials. The rationale behind ADCs, as well as their primary features and recent research breakthroughs, will be discussed. We then offer an approach for maximizing the potential value that ADCs can bring to cancer patients by highlighting key ideas and distinct strategies.

## Introduction

Cancer therapy remains a difficult task. Chemotherapy has a significant clinical benefit for many tumours, but it has low selectivity and high toxic effects, result in devastating effects and decreased therapeutic efficacy [1]. Antibody–drug conjugates (ADCs) are a promising cancer treatment that includes delivering toxic drugs to specific tumor cells that exhibit specific antigens connected to malignancy. The antibody, cytotoxic agent, and linker are the three primary structural units of an ADC. ADCs are expected to provide powerful therapeutic modalities against various cancers by combining the selectivity of monoclonal antibodies (mAbs) and the efficacy of various chemotherapeutics [2]. Together, the three components comprise a highly effective anti—tumour agent directly and selectively providing chemotherapy drugs to cancer cells, directed by antibodies with exceptional specificity and affinity.

Cleavage of the ADCs linker components by certain tumor-associated enzymes (i.e. matrix metalloproteinases) or by lower pH encountered in the tumour microenvironment results in the release of the active component [3]. These non-internalizing ADCs did not increase drug selectivity and, as a result, did not reduce toxicity considerably [4]. Despite the fact that ADCs have been studied for many years, we have only just recognized their true potential, thanks to significant advancements in linker and conjugation technology, as well as very powerful cytotoxic drugs [5]. ADCs are intended to broaden the therapeutic window of these medications by only delivering them to tumour cells that express a specific antigen targeted by the ADC’s mAb antigen [6, 7]. The properties of the antibody, therapeutic payload, and linker are critical in the overall efficacy of ADCs, which is dependent on intricate interactions between the ADCs and numerous tumour cell and tumour microenvironment (TME) targeting components [8].

Despite promising ADC-induced therapeutic activity against resistant and recurrent cancers, several barriers remain to their widespread use, including unidentified drug resistance mechanisms, toxicity, the lack of predictive prognostic biomarkers, and their clinical advantages over standard therapies. The development of new ADCs is a continual process that relies on advancements in several technologies such as biosynthesis of novel linkers, mAb synthesis and manufacturing, and the introduction of new payloads that are more powerful against tumour cells with fewer systemic side effects.

### History, design, construction and mechanism of action of ADC

In the twenty-first century, the development of ADCs has reached significant milestones. Since the early 1900s, efforts have been made to improve the safety and efficacy of Paul Ehrlich’s “magic bullet”, which was the first therapeutic technique to convey lethal drugs to selected cancer cells depending on the presence of cell specific antigen(s) [9, 10]. Leukemia cells were targeted after the successful chemical linkage of polyclonal rodent immunoglobulins and methotrexate [11]. Hybridoma technology permitted the manufacturing of mAbs in 1950, and by the early 1970s, it had sparked important breakthroughs in the field of ADCs, both in vitro and in vivo [12].

The use of ADCs in animal models was described in the literature in the 1960s, and clinical trials with ADCs based on mouse immunoglobulin G (IgG) molecules were conducted in the 1980s [13].

The first ADC to be approved by the US Food and Drug Administration (FDA) for the treatment of patients with acute myeloid leukaemia was gemtuzumab ozogamicin (developed by Wyeth).

This was followed by the approval of two second generation ADCs: brentuximab vedotin (developed by Seattle Genetics) in 2011 [14, 15] and trastuzumab emtansine (also known as T-DM1 and ado-trastuzumab emtansine; developed by Roche) in 2013 [16], both of which target the cancer antigens CD30 (also known TNFRSF8) and human. Since 2013, the field has changed dramatically. More than 30 new ADCs have entered clinical development (all for oncological indications), and more than 60 ADCs are currently in clinical trials [17]. A list of the most recent ADCs currently approved by the US FDA is provided by Drago el al [18, 19].

Clinical trials for treating cancer patients with ADCs began in 1980, however the trials’ clinical usefulness was hampered by the development of medication toxicity without a significant clinical benefit [20–22]. Gemtuzumab ozogamicin, an FDA approved CD33-targeted medication for the treatment of relapsed and/or refractory acute myeloid leukemia (R/R) has been withdrawn from the market due to unfavorable adverse effects (AE) [23–25]. Brentuximab vedotin, a CD30-targeted ADC, and ado-trastuzumab emtansine (T-DM1), a HER2-targeted ADC, were approved in 2011 and 2013, respectively, for the treatment of R/R classical Hodgkin lymphoma and trastuzumab-resistant metastatic breast cancer [15, 26, 27]. Several ADCs are currently being studied in preclinical and clinical development, and the FDA has fully approved nine of them [28].

### Components of ADC: mAb, linker, and payloads

**Fig. 1:**
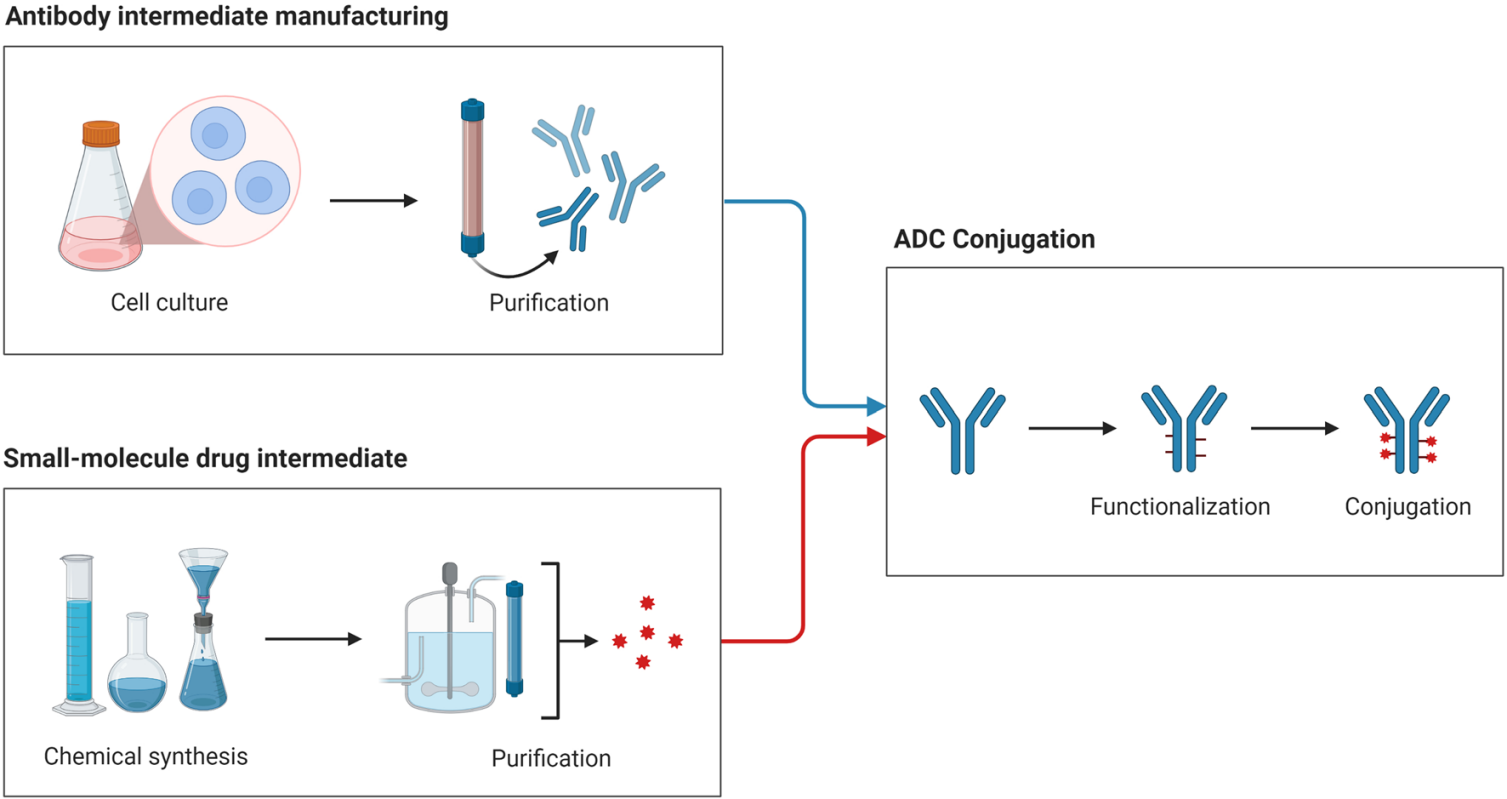
The development, purification, and production of antibody-drug conjugates

ADCs are primarily composed of three major components: a drug, a linker, and an antibody. The efficacy of each ADC is largely determined by differences in the three fundamental components of ADCs. The development and purification of mAbs utilizing proper cell culture techniques are among the phases in the production of antibody–drug conjugates. Chemically generated and refined cytotoxic payloads. After being functionalized using a specific linker, the mAbs are finally attached to the cytotoxic drug payload (Fig. 1).

### Antibodies selection

In addition to cancer treatment, antibody-based therapies have made significant advances in the treatment of other diseases including autoimmune diseases, and cardiovascular and bone diseases [29]. One of the most crucial parts of ADC design is antibody selection,and high antigen specificity [30]. Antibodies with low specificity that cross-react with other antigens might have unpredictable effects, by interacting with healthy tissues, it might cause off-target toxicities or cause premature clearance from the body before it reaches the tumor site [30]. Immunoglobulin M (IgM), A, D, IgE, and IgG are the five types of antibodies. Among the five immunotherapy classes, IgG is the most commonly used [31]. Despite the enormous potential for innovation offered by antibody fragments and bispecific antibodies, immunoglobulin G (IgG) is currently the most common used in in ADCs [32–34]. The classical complement system is activated by IgG subclasses, particularly IgG1 and IgG3. The membrane attack complex (MAC) forms pores on the tumour cell surface leading to cancer cell lysis [35]. IgG1 antibodies have similar serum half-lives to their IgG2 and IgG4 counterparts, but higher complement-fixation and FcR-binding efficiency. Although IgG3 antibodies are the most immunogenic, they are often avoided in ADC design due to their short circulation half-lives [36]. The immunogenicity degree of an ADC is a critical aspect that influences circulatory half-life [7]. mAbs can penetrate tumor after being administered into the bloodstream [2]. The antibody’s size, which typically accounts for roughly 95% of an ADC's bulk, prevents ADCs from spreading into tumor tissue.

### mAb target selection

Searching for cell-surface proteins expressed in tumours rather than non-malignant tissues has been one guiding methodology in selecting the right mAb target [37]. HER2, TROP2, and Nectin 4 are effective targets for ADCs now approved for the treatment of solid malignancies [38–40]. With the exception of a tiny fraction of lymphocytes, CD30 is a target of brentuximab vedotin and is expressed by malignant lymphoid cells in Hodgkin lymphoma and ALCL in the setting of haematological malignancies [41]. Similarly, inotuzumab ozogamicin, polatuzumab vedotin, and belantamab mafodotin are highly specific for hematological malignancies lineages [42, 43].

Different mAbs may have different Fc-dependent effector activities [44]. As a result, mAbs designed for other therapeutic uses may not be the optimal ADC backbones, in particular when considering mounting evidence that internalization and intracellular trafficking of ADC are critical to ADC cytotoxicity. Pertuzumab’s affinity for HER2 is pH-dependent, unlike trastuzumab, resulting in rapid dissociation of the Ab–Ag complex in a low-pH environment. As a result of this discovery, a preclinical recombinant pertuzumab-based ADC with increased cytotoxicity was developed [45]. Variant proteins are more prone to ubiquitylation, absorption, and/or instability than wild-type counterparts when targeted by ADCs [46, 47]. ADCs based on mAbs that target proteins with mutations of truncal oncogenic driver (for instance some mutant versions of EGFR) could attain tumour specificity levels hitherto only achieved with extremely selective inhibitors of small-molecule tyrosine kinase [48, 49]. Bispecific antibodies have opened up new research and development opportunities. Antibody absorption and/or processing, but also tumor selectivity, could all benefit from such compounds [50].

### Linker design and technologies

The specificity, efficacy, and safety of an ADC are determined by the design, structure, and chemistry of the linker that connects the cytotoxic payload to the antibody. Linkers are typically designed to be constant in the blood system (enabling for a prolonged timeframe of bloodstream), but labile enough to efficiently deliver the cytotoxic payload to the tumour [51].

Cleavable and non-cleavable linkers are the two types of linkers. Following exposure to acidic or reducing environments or proteolytic enzymes, cleavable linkers are cut and release the ADC’s cytotoxic payload (for example, cathepsins). pH-sensitive hydrazone (found in brentuximab vedotin, enfortumab vedotin, polatuzumab vedotin, trastuzumab deruxtecan, and sacituzumab govitecan) is another enzyme-cleavable peptide-based linker (T-DXd) [51, 52].

**Fig. 2:**
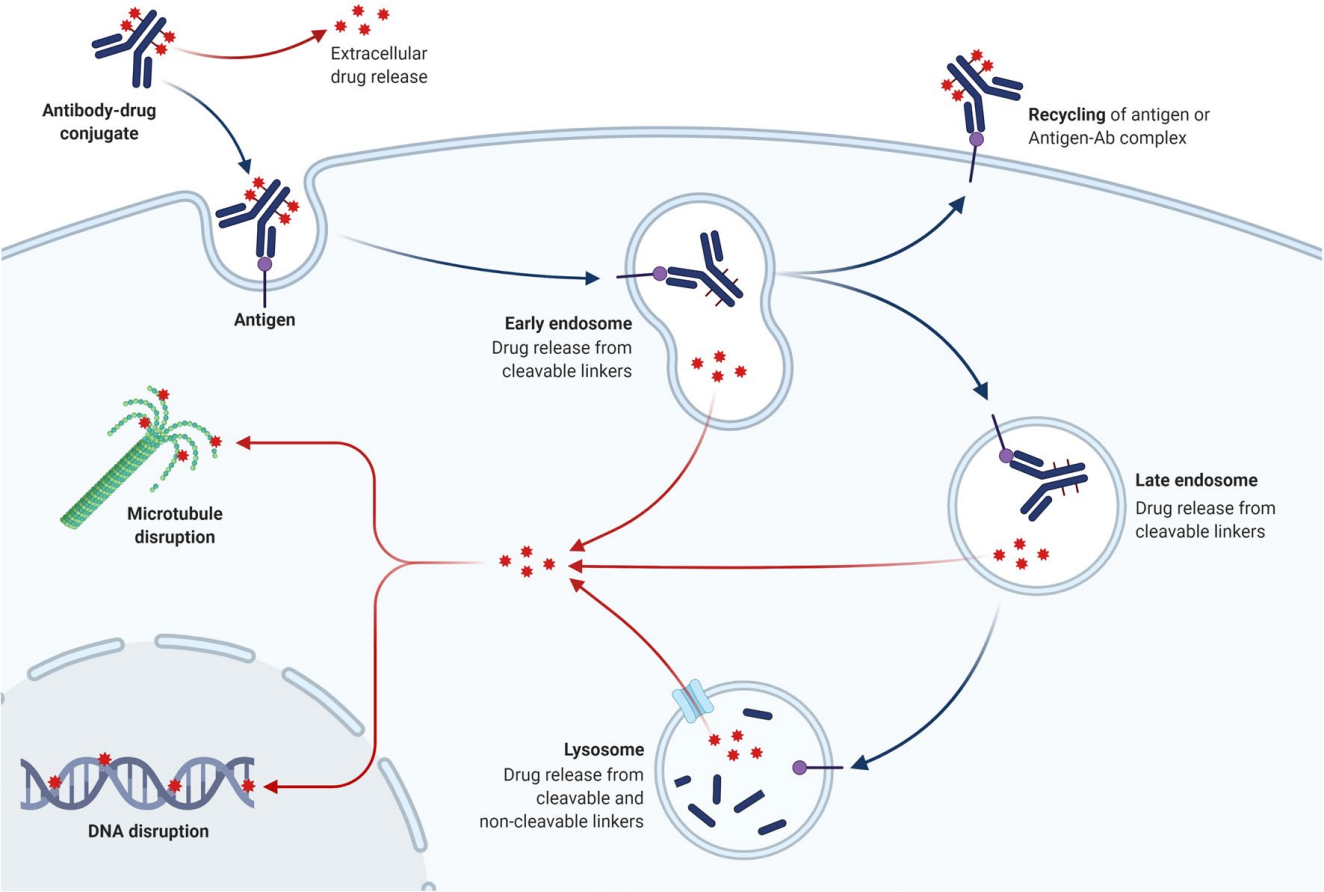
ADC internalization and destruction of both the antibody and the linker

Non-cleavable linkers are becoming more appealing than cleavable linkers due to the advantage of greater plasma stability. Furthermore, studies show that non-cleavable linkers perform far better in vivo, with payload release occurring primarily in the lysosome following ADC internalisation and destruction of both the antibody and the linker (Fig. 2). As a result, the danger of systemic toxicity from premature payload release is reduced. As a result, non-cleavable linkers may offer a wider therapeutic window, as well as increased stability and tolerability [53]. T-DM1 with mafodotin belantamab is one of two FDA-approved ADCs with non-cleavable linkers [18].

The systemic stability of ADCs post administration is one of the crucial issues to ensure the efficacy of ADCs, and several strategic approaches are taken for overcoming this issue. ADCs should ideally remain stable or intact in the circulation before entering target cells, but there are cases where ADC catabolites are still biologically active [54, 55]. ADC stability refers primarily to metabolic stability or integrity. To improve ADC stability, several approaches involving conjugation site selection and linker modification have been developed [54]. In general, modifications to each component (e.g., antibody, linker, and payload) can be performed for this purpose. The conjugation site, linker length, and linker steric hindrance are effective general approaches for site-specific ADCs and should be more broadly applicable to a variety of ADC platforms [54]. By choosing a more sterically hindered conjugation or attachment site, the antibody can provide the desired steric shield. On the other hand, introducing proximal steric hindrance around the cleavable or labile site of the linker has been shown to be an effective method of improving stability [56]. ADC biotransformation and drug-antibody-ratio (DAR) profiling have evolved into critical integrated data for assessing and comprehending ADC stability [54, 57].

### Payloads

Monoclonal antibodies (mAbs) are well-known therapeutic agents used to treat a wide range of illnesses, including cancer [58]. Because of the limitations of mAbs’ anticancer activity, researchers are working to improve their potential efficacy. These efforts include mAb conjugation to radionuclides, fusion with immunotoxins, and coupling to ADCs. Payload [59] is the combination of a mAb with a cytotoxic agent or a small molecule. Methotrexate, doxorubicin, and vinca alkaloids are examples of traditional chemotherapy drugs with proven anticancer activity [21, 60, 61], were initially carried by ADCs. ADCs sometimes required high dosages to be effective, as result of increasing systemic toxicities [62]. Currently, optimizing ADCs is a never-ending problem, with most research and development activities focusing on the mAb or chemical linker, on small-scale endeavors, aimed to optimize the cytotoxic payload. There is a dearth of diversity in the medicinal payloads used in the 114 finished or continuing human trials, with only 7 payload formulations described (4 additional ongoing clinical studies with undetailed structures). Natural products account for six of the seven payload mixes, emphasizing the importance of natural materials as cytotoxic payloads for ADC in research investigations [63]. Furthermore, the findings demonstrate that a small part of the mAbs targeting the tumor (on the order of 0.1 percent) penetrates tumor tissue, emphasizing the significance of larger cytotoxicity payloads for treatment response [64, 65]. These discoveries contributed to the growth of ADCs, which include highly effective chemotherapeutic medications like as auristatins, calicheamicins, camptothecin, and maytansinoids analogs that can be lethal even at sub-nanomolar quantities [66, 67]. Nine cytotoxins were generated from plants, and 21 were natural product formulations from 79 anticancer and antiviral approved medications, according to FDA investigation from 1983 to 2002 [68]. Furthermore, 13 of the 39 anticancer compounds were based on natural chemicals. Sixty percent of contemporary pharmaceuticals are bioengineered from natural sources [68, 69].

For determining ADC efficacy, the drug–antibody ratio (DAR), or the amount of drug molecules attached to a single ADC, is critical. DAR varies a lot and is influenced by other ADC variables [70]. The DAR values are also affected by the conjugation site and whether light or heavy conjugated chains are used [70]. The DAR value affects the medicine’s effectiveness since low drug loading reduces potency, whereas high drug loading can affect toxicity and pharmacokinetics (PK) [71, 72]. In general, there are two types of payloads that are commonly utilized in ADC design, as listed below.

Rapid plasma clearance may limit the ability of small-molecule drug conjugates to reach tumor cells or poorly vascularized tumors or the central nervous system [73–75]. Other innovative ADCs include immunostimulatory agents such Toll-like receptor agonists, chemokines, or STING agonists to attract and/or activate immune effector cells to tumours [76, 77]. Several ADCs containing cytotoxic radioisotopes, notably the CD20-targeted drugs ibritumomab tiuxetan, 131I-tositumomab, and 131I-rituximab [78], have shown clinical activity against lymphomas. Prostate cancer (trying to target prostate-specific membrane antigen), glioblastoma (directly attacking EGFR), and gastrointestinal tumors (designed to attack carcinoembryonic antigen) are all being studied with similar treatments [79]. Antibodies can transport oligonucleotides, allowing for in vivo selective modification of signal transduction pathways [31].

### Microtubule-disrupting agents

The synthetic antineoplastic agent auristatin is produced from dolastatin 10, a natural substance [80]. Because dolastatin 10 is a nonspecific toxic chemical, it is not used as a cytotoxic warhead in ADCs. In this class of drugs, synthetic analogues including MMAE and MMAF are currently being employed in ADCs as a cytotoxic payload [81]. MMAE is an antimitotic drug that works by preventing tubulin polymerization, which causes cell cycle arrest and apoptosis [82].

Maytansinoids are a second significant family of microtubule-disrupting drugs derived from the benzoansamacrolide maytansine. Tubulin polymerization is inhibited by these medications, resulting in mitotic arrest then cell death [83]. Maytansinoids perform the same action as Vinca alkaloids. The cytotoxicity of the maytansinoids, on the other hand, was over 100 times that of the Vinca alkaloids [84]. Maytansinoids have failed in human trials as anticancer treatment due to a lack of tumour selectivity and substantial systemic toxicity. Maytansinoids’ potent cytotoxicity can be used as a targeted delivery vehicle, notably in the form of antibody–maytansinoid conjugates (AMC).

### DNA-damaging agents

Calicheamicins are a kind of enediyne antitumor antibiotic produced from the *Micromonospora echinospora* bacterium [85]. Calicheamicin binds to the minor groove of the TCCTAGGA DNA sequence and prevents it from replicating [86]. The payload in the ADC design is N-acetyl-calicheamicin, a calicheamicin derivative [87]. Gemtuzumab ozogamicin, sometimes known as Mylotarg, is the name of this ADC. It consists of a humanised IgG4 mAb conjugated to a calicheamicin payload that targets the CD33 surface antigen, which is present in 85–90% of individuals with acute myeloid leukaemia [88].

Duocarmycin is a natural chemical generated from bacterium strains of the Streptomyces genus [89]. Duocarmycin is another DNA minor groove–binding alkylating agent. By binding to the minor groove of DNA and causing persistent alkylation of DNA, this family of medicines affects nucleic acid architecture and thus structural integrity [90]. Yu and colleagues' work recently highlighted an example of duocarmycin application in ADC setting [91]. Promiximab-DUBA, a new ADC against CD56, was described in this work. In this ADC, an anti-CD56 hIgG1 antibody is linked to the payload duocarmycin via a reduced interchain disulfide linker. In vitro and in vivo, this novel ADC showed significant cytotoxic effect against cancer cells.

Doxorubicin works by intercalating DNA, which prevents DNA synthesis [92]. One well-known example of doxorubicin-based ADC design is the milatuzumab-conjugated doxorubicin ADC (IMMU-1010), which has been employed in phase I/II clinical studies for the treatment of CD74-positive relapsed multiple myelomas [93].

### Mechanism of action of ADCs

**Fig. 3:**
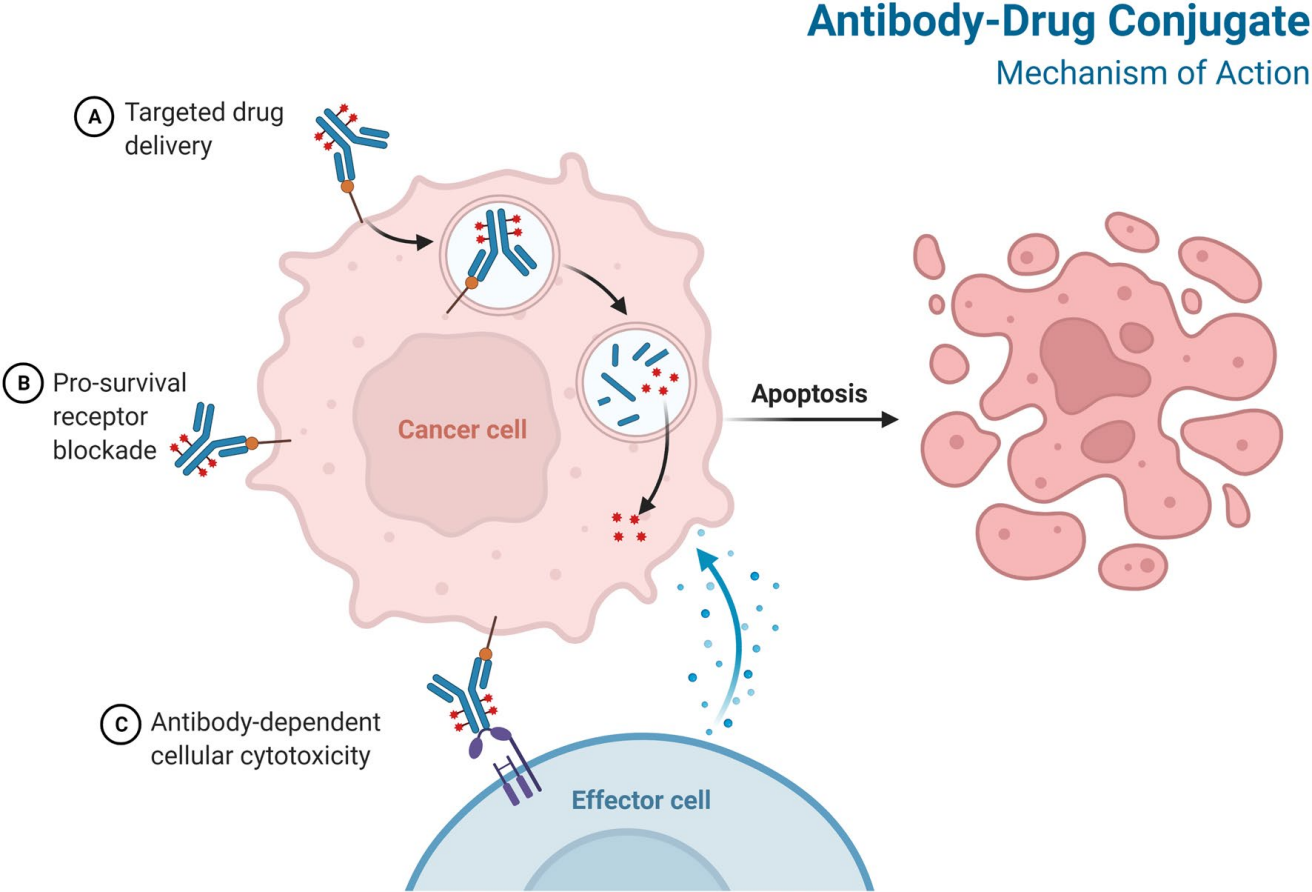
Mechanism of action of ADC

Preclinical studies are conducted to determine a starting dose for human trials as well as to assess the product’s toxicity. Antibody/antigen binding studies, in vitro cytotoxicity testing, in vivo anti-tumor efficacy studies, pharmacokinetics, and toxicological studies in rodents and nonhuman primates should all be included in the preclinical evaluation of ADCs [94]. ADCs are given intravenously into the circulation to avoid stomach acid and proteolytic enzyme degradation of the mAb [95]. The mAb component of ADCs must be expressed selectively on tumor cells and not on normal cells in order to identify and bind to the target antigens [96] (Fig. 3).

ADCs combine antibody and cytotoxic drug activities to provide various modes of action and pharmacokinetic properties [18]. The complexities of ADC activity in the clinic are only now becoming recognized [97]. The antigen-ADC complex is absorbed by receptor-mediated endocytosis (clathrin- or caveolae-mediated endocytosis) or pinocytosis [98, 99] (Fig. 3). Internalization causes the cell membrane to bud inward, resulting in the formation of an early endosome, which grows into a late endosome before joining with lysosomes [100]. The antigen-ADC complex is then broken down [100]. Antimicrotubular medications, for example, are small compounds that can cross the lysosomal membrane and into the cytosol [101]. In some cases, the cytotoxic payload may be ejected from the cell or discharged into the tumor microenvironment after being liberated in the cytoplasm. Bystander death occurs when the cytotoxic payload harms cells that do not display the target antigen [102, 103]. This effect is influenced by a number of factors, including the type of linker employed and the properties of the payload, although the presence of cell-permeable payloads enhances the effect [72]. The cell-killing path is determined by the payloads used. Cell death is caused by auristatins and maytansinoids interfering with microtubulins, whereas cell death is caused by calicheamicins and duocarmycins intercalating DNA [7]. The ability of an ADC to produce cytotoxicity is determined by a number of factors, including the characteristics of the target antigen's properties, the choice of an antibody, the creation of a stable linker, and the conjugation of effective payloads.

Antibodies reach tumour cells by passive diffusion after leakage from capillaries, which results in sluggish and inefficient absorption [104–107]. The Fab and Fc portions of ADCs have an influence on antibody-dependent cellular cytotoxicity (ADCC), complement-dependent cytotoxicity, and antibody-dependent cellular phagocytosis [108, 109]. T-DXd and T-DM1, for example, share the same ADCC-competent IgG1 backbone and elicit ADCC in vivo, implying that ADCs could be used as immunotherapy [41, 110]. Antigen-dependent endocytosis or antigen-independent pinocytosis can be used to internalize ADCs, with clathrin-mediated endocytosis being the most prevalent [111–114]. Early endosomes are more likely to release payloads with acid-cleavable linkers, whereas late endosomes or lysosomes are more likely to release payloads with enzymatically cleavable linkers [111]. Certain ADCs can have a ‘bystander effect’ on neighboring cells, regardless of the compartment into which the payload is delivered [72].

### Mechanism of resistance against ADC

The failure or ineffectiveness of a treatment is defined as drug resistance. Such failure/reduction may have arisen as a result of drug therapy (secondary or acquired resistance), or it may have existed from the start of treatment (primary or de novo resistance). Resistance to ADCs might theoretically be equivalent to resistance to the ADC’s individual components, namely the mAb and the cytotoxic agent. Despite the need for more research, existing clinical data reveal that patients who develop trastuzumab resistance with a taxane react to T-DM1 [115], implying that T-DM1 action is unrelated to previous therapy lines such as anti-HER2 medications or chemotherapies.Because ADCs are targeted therapeutics, fluctuations in the antigen levels detected by the mAb could be a source of resistance. Loganzo and colleagues [116], for example, used many cycles of anti-HER2 trastuzumab–maytansinoid ADC therapy to generate a variety of T-DM1-resistant breast cancer cell lines. Gemtuzumab ozogamicin is consumed by high amounts of CD33 in the blood (GO), [117]. For HER2, truncation of the antigen’s ectodomain or the masking by components of the extracellular matrix have been proposed as mechanisms of trastuzumab resistance [118]. However, masking or shortening of the epitope as mechanisms of ADC resistance have yet to be reported in preclinical animals.

A common mechanism of chemotherapeutic resistance is the clearance of the medication from the cellular cytoplasm via ATP-binding cassette (ABC) transporters [119]. Because many cytotoxic medicines are ABC transporter substrates, ADC resistance may be caused by these drug efflux pumps [120]. In preclinical studies, PgP/MDR1 expression was observed in AML cells that were resistant to GO [121].

One recently reported mechanism of T-DM1 resistance is the drug’s action on cyclin B, a cell-cycle protein involved in the G2–M transition. T-DM1 produces a rise in cyclin B in HER2 + breast cancer cells that are sensitive to the treatment, but not in cells that are resistant to the drug ^122^. Furthermore, suppressing cyclin B led in drug resistance. T-anticancer DM1’s effect coincided with cyclin B buildup in a patient cohort of 18 HER2 + breast cancer fresh explants. These findings have clinical implications since cyclin B induction could be utilized as a biomarker for T-DM1 sensitivity.

Activation of downstream signaling pathways can lead to resistance to ADCs. In primary AML cells, GO resistance has been associated to increased PI3K/AKT activation in vitro. In this study, MK-2206, an AKT inhibitor, dramatically sensitized resistant cells to GO or free calicheamicin [123]. In a clinical trial, the safety and early evidence of the efficacy of combining T-DM1 and a PI3K inhibitor are being studied (Clinical trials identifier: NCT02038010). ADC sensitivity may be influenced by variations in apoptosis regulation. BAX and BAK, two pro-apoptotic proteins, have previously been linked to the regulation of GO sensitivity in AML [124].

### Resistance-breaking and ADC-based therapy optimization strategies

Resistance to ADCs has been one of the problems limiting these medications’ clinical success. ADCs’ modular nature allows for the modification of some of its components in order to create novel compounds able to overcome resistance. Increased expression of drug efflux pumps is one of the most common mechanisms of ADC resistance. Changing the cytotoxic agent for medicines or poisons that are poor efflux substrates is one way to get around this. In AML animal models, for instance an anti-CD33 antibody conjugated to PBD, vadastuximab talirine, showed strong effectiveness, even in those where GO had little effect [125].

A second option is to change the linker’s hydrophilicity, that can diminish MDR because MDR1 transports hydrophobic chemicals more efficiently than hydrophilic substances. Polar linkers such as sulfo-SPDB (58) and mal-PEG4-N-hydroxysuccinimide have showed enhanced effectiveness against MDR1 + animals [120].

To improve ADCs, the linker-cytotoxic structure can be changed [126]. Because tumour heterogeneity is a major problem in cancer, ADCs may be unable to destroy low-antigen–expressing cells.

Resistance could also be overcome by new mAb forms, such as bispecific or biparatopic ADCs. In the case of HER2, this has been demonstrated. The first biparatopic ADC, which targeted two nonoverlapping HER2 epitopes, was demonstrated to cause HER2 receptor clustering, which resulted in significant internalization and degradation, as well as anticancer activity in T-DM1–resistant tumor models [50]. This biparatopic ADC is now being tested in a number of phase I studies in patients who have failed or are ineligible for HER2-targeted therapies.

Finally, it appears that combining ADCs with other immunotherapies is a promising strategy [127]. The addition of ADCs to immune checkpoint inhibitors may improve treatment response by increasing the recruitment of CD8 + effector T lymphocytes to tumor tissues.

### ADCs toxicities

Several initial tests of ADCs revealed significant adverse effects (AEs) [49]. ADCs were created with the primary purpose of increasing tumor targeting and decreasing the toxicities caused by conventional chemotherapy drugs. Surprisingly, cardiac toxicity appears to be rarer with HER2-targeted ADCs than with trastuzumab that has not been conjugated (although the frequency of those AEs still requires an appropriate monitoring). The reason for this apparent difference is unknown; higher cardiotoxicity could be expected if an ADC delivered a cytotoxic payload directly into HER2-expressing cardiomyocytes, but this effect has never been demonstrated in clinical studies. Payload release in the bloodstream, non-malignant tissues, or the TME, as well as the payload's following effects in non-tumour tissues, could be implicated for off-target toxicities [128]. The target antigen’s expression pattern influences the cytotoxic drug’s distribution, which can sometimes result in serious “on-target, off-tumor” toxicities which are not always payload related. In the early 1990s, the ADC BR96-doxorubicin was seen to be highly efficient in mouse xenograft models of a variety of tumor types; however, unlike mice, this antigen is found in non-malignant organs in humans, particularly the gastrointestinal system. T-DXd and trastuzumab duocarmycin, two HER2-targeted ADCs with distinct payloads, both cause pulmonary toxicity through an unknown mechanism [129].

Many ADCs may detect the target antigen in non-tumor tissues, but not at sufficient levels to cause damage. Other proteins, such as TROP2, the sacituzumab govitecan target, are expressed in a number of non-malignant organs, but they are only accessible if they are abnormally expressed on the surface of some tumor cells [50, 51].

### Strategies to improve ADCs efficacy in clinics

Tumors that have been heavily pre-treated have a lot of genetic instability, which causes inter- and intra-tumoral heterogeneity, as well as hypoxic and immunosuppressive TMEs that limit medication penetration [130].

Enfortumab vedotin, a nectin 4-targeted ADC with an MMAE microtubule inhibitor payload, achieved a 44 percent ORR in patients with urothelial metastatic carcinoma previously treated with platinum-based chemotherapy and immune-checkpoint inhibitors [131].

In patients with advanced-stage HER2-positive breast cancer who had received prior T-DM1 and five additional regimens, T-DXd, a next-generation HER2-targeted ADC, achieved an ORR of 60.9 percent (36). T-DM1 is a combination of trastuzumab anti-HER2 antibody and a microtubule-targeting payload (DM1) that has showed promise in patients who have developed resistance to trastuzumab and other microtubule-targeting chemotherapies such as taxanes and vinca alkaloids. T-DXd, which contains a TOPO1 inhibitor, has shown clinical activity (ORR 51%) against gastrointestinal cancers that are only moderately responsive (ORR 14%) to irinotecan, another TOPO1 inhibitor [132, 133]. As previously stated, intratumoural heterogeneity is a primary cause of targeted therapeutic resistance [134]. The bystander effect appears to boost the efficiency of ADCs with cleavable linkers and membrane-permeable payloads, emphasizing chemotherapy’s indiscriminate cytotoxicity targeting antigen-negative cells in close proximity to antigen-positive cells [71, 135].

There is an obvious need for improved predictive biomarkers to direct ADC therapy [52, 136]. IHC is the principal approach for measuring the expression of target proteins. IHC, on the other hand, is at best a semi-quantitative assay, and a number of cut-offs have been used to define target positive without explanation. Cytotoxicity may or may not be proportional to targeted antigen expression after a particular density of a certain cell-surface antigen is achieved for ADC activity [6]. Meanwhile, some basic concepts can be used to guide the combination of an ADC with a certain cancer type in order to improve treatment success. We anticipate that stable ADCs with non-cleavable linkers will be most beneficial in situations where the target antigen is highly and uniformly overexpressed in a tumor-specific manner [137]. This method is likely to effectively eliminate cancer cells while minimising systemic side effects. Labile and/or cleavable ADCs, on the other hand, are expected to rely on the bystander effect to overcome tumour heterogeneity or low-level target expression, often at the expense of off-target effects [71, 138]. Using irreversible kinase inhibitors against the ADC target concurrently (for example, neratinib with HER2-targeted ADCs) can increase antigen internalization and thus ADC endocytosis and activity [47]. ADCs are now being evaluated in over 20 clinical trials in conjunction with approved or investigational immunotherapies. This method is founded on the idea that ADC-mediated cell death and tumor-infiltrating lymphocyte recruitment assist immune effector cells in recognizing immunologically 'cool' tumors and/or improving ADC function.

### Inflammatory responses against ADCs

Of the 110 mAb preparations currently approved by the FDA and/or EMA, 46 (including 13 antibody–drug conjugates) recognise 29 different targets for cancer treatment, and 66 recognise 48 different targets for non-cancer disorders. An updated recent list of FDA approved ADCs against various cancers are provided by recent reviews [18, 126]. Despite their specific targeting and the expected reduced collateral damage to normal healthy non-involved cells, mAbs can cause type I (anaphylaxis, urticaria), type II (e.g., hemolytic anaemia, possibly early-onset neutropenia), type III (serum sickness, pneumonitis), and type IV (Stevens-Johnson syndrome, toxic epidermal necrolysis) hypersensitivities, as well as other [139]. The release of a cascade of cytokines associated with inflammatory and immunological processes is a feature shared by the majority of these syndromes. Antibodies targeting the epidermal growth factor receptor may cause non-immune papulopustular and mucocutaneous eruptions.

## Conclusions

ADCs are a novel cancer treatment that combines pharmacogenetic testing with tailored medication. They successfully minimise the systemic toxicity of chemotherapy and provide novel therapeutic alternatives for diseases with poor prognoses and few treatment options. ADCs could be a potential therapy option where specific targeting by antibodies is possible and cell death of the target is the therapeutic goal. ADCs are unique, potent, and unpredictable, and clinical and translational researchers are only beginning to grasp them. Overall, a better understanding of ADC processing and the events that happen after antibody-antigen contact would be tremendously valuable to the ADC development field T. The potential of such a pharmacological platform for cancer treatment could be far-reaching and potentially transformative if the complexity of ADC–tumor interactions can be better understood and utilized. The development of next-generation ADCs with site-specific linker technology, enhanced mAb selectivity, and more effective cytotoxic payloads is presently underway, as are clinical trials to determine the optimum ADC dosage strategies.

## Data Availability

All data are available in the manuscript.
